# Effects of intravenous laser irradiation on viral load and T lymphocytes in people living with HIV/AIDS

**DOI:** 10.1590/1980-220X-REEUSP-2025-0004en

**Published:** 2025-09-05

**Authors:** Fabiana Tomé Ramos, Maria Cecília Yoshioka Lobo, Hélio Amante Miot, Eliana Maria Minicucci, Rúbia Aguiar Alencar

**Affiliations:** 1 Universidade Estadual Paulista, Faculdade de Medicina, Botucatu, SP, Brazil.

**Keywords:** Low-Level Light Therapy, HIV, Acquired Immunodeficiency Syndrome, Lymphocyte Count, Viral Load

## Abstract

**Objective:**

To evaluate the effectiveness of intravenous laser irradiation of blood in reducing viral load and increasing LT-CD4+ and LT-CD8+ in people living with HIV/AIDS.

**Method:**

Randomized, controlled, parallel, single-blind clinical trial. Twenty-eight participants were allocated to the intervention (ILIB *n* = 15) and control (CTRL *n* = 13) groups. The ILIB group received irradiation with red laser (660 nm), applied to the radial artery (30 minutes/day for 20 days), with a total energy of 180 J. The CTRL group received a sham intervention (without light emission). Viral load and LT-CD4+ and LT-CD8+ counts were assessed at the end.

**Results:**

Both groups showed a reduction in viral load (ILIB: −1.7 log; CTRL: −2.3 log). In the ILIB group, 61.5% of participants achieved an undetectable viral load (p = 0.449). There was no increase in CD4+ LT count (p = 0.287), but a non-significant reduction in CD8+ LT count was observed in the ILIB group (p = 0.173). There were no statistically significant differences between groups for any outcome (p > 0.17). The study was stopped prematurely during the interim analysis.

**Conclusion:**

ILIB, as an adjuvant therapy to ART, did not show clinical or statistical superiority in the immunological and virological parameters evaluated.

**Brazilian Registry of Clinical Trials (ReBEC):**

RBR-7j9rv5d (https://ensaiosclinicos.gov.br/trial/10848)

## INTRODUCTION

Although there are promising therapies associated with the cure of HIV, there is not enough evidence to support this. There is still no cure for HIV infection, but current antiretroviral therapy (ART) is effective. The goal of treatment is to inhibit the replication of the virus in the body; preserve/restore the immune system; reduce the likelihood of the emergence of more resistant viral strains; decrease morbidity, mortality and hospitalization rates; decrease the risk of developing opportunistic diseases and increase the length and quality of life of people living with HIV/acquired immunodeficiency syndrome (AIDS) (PLWHA)^(1)^. This treatment has a major impact on PLWHA survival, thus altering the epidemiological characterization of HIV/AIDS^(1)^.

The Clinical Protocol and Therapeutic Guidelines for the Management of HIV Infection in Adults from the Ministry of Health states that the objective of monitoring and treatment is to achieve an undetectable viral load (VL)^(2)^. However, even with ART and monitoring, some PLWHA do not reach this level, which may occur due to poor adherence to treatment and viral resistance^(3)^.

A suggested strategy to achieve undetectable VL in PLWHA is the use of intravenous laser irradiation of blood (ILIB), as an adjuvant therapeutic alternative.

Lasers, since their creation, have undergone great technological evolution in the areas of physics and medicine, being applied in the treatment of various pathologies. They consist of an active medium—solid, liquid, or gas—that emits light when excited by an energy source^(4)^. The introduction of low-level laser therapy, in 1966, was consolidated as the most investigated therapeutic modality in the field of clinical applications of laser^(4)^.

Originally, ILIB was performed through venipuncture to which the device was attached. Currently, with technological advances, it can be done transcutaneously, close to the blood vessels, that is, in contact with the skin, without the need for puncture^(5)^. Thereafter, in 2021, it was suggested that the term used to describe this non-invasive procedure should be “vascular photobiomodulation”^(6)^. However, there is still divergence in the literature regarding the terminology used, with studies referring to it as “transcutaneous ILIB”^(7)^, “modified ILIB”^(8)^, “vascular systemic photobiomodulation”^(9)^, among others. Thus, in the present study, considering that the term ILIB continues to be used frequently, we chose to refer to the technique as ILIB, in a more generalized way.

ILIB works by modulating the immune response, directly stimulating antioxidant enzymes such as superoxide dismutase (SOD), catalase, and glutathione peroxidase^(10)^. These enzymes have the function of neutralizing free radicals present in the body, reducing oxidative stress, which is a common factor in several inflammatory and infectious conditions^(10)^.

In people living with HIV, oxidative stress is intensified by the continued presence of the virus, which can contribute to disease progression and the emergence of comorbidities^(11)^. Therefore, ILIB can act as an adjuvant therapy by modulating the immune system and reducing oxidative stress. This systemic effect may favor the preservation of CD4+ T cells and improve the immune response, contributing to the reduction of viral progression and associated comorbidities.

Clinical studies show positive effects of ILIB in regulating blood pressure, glucose, triglycerides and cholesterol^(12)^, improvement of thrombocytopenia, neutropenia and hemoglobin disorders in chemotherapy patients^(13)^, protection against ovarian aging, improvement of independence of post-stroke patients^(14)^, improvement of symptoms in people with long-term COVID-19^(15)^, pain and sleep disorders^(16)^, among others.

However, only one study was found using ILIB in PLWHA. In this study, ILIB was used in the regions of the cubital vessels, liver and thymus, showing a reduction in the signs and symptoms of PLWHA and hepatitis, an increase in the counts of T-CD4+ lymphocytes (LT-CD4+), T-CD8+ lymphocytes (LT-CD8+), and total lymphocytes and a decrease in VL in participants with HIV infection^(17)^. This study was carried out from 1995 onwards on people who were not yet using ART. After 1998, ILIB was introduced as a treatment for some complications of ART, such as nausea, intoxication and impairment of general health, presenting positive effects and an increase in leukocyte count^(17)^.

The authors of the study believed that laser irradiation could interfere with the cell cycle altered by the infection, leading to increased blood microcirculation and the regeneration of damaged cells. It was also observed that the plasma viral load remained low for a long time after the end of treatment with ILIB^(17)^.

Given the scarcity of studies on ILIB in PLWHA, the present study aimed to evaluate the effectiveness of ILIB in reducing VL and increasing LT-CD4+ and LT-CD8+ counts in PLWHA. The study hypothesis was that PLWHA undergoing ILIB would present a reduction in VL and an increase in LT-CD4+ and LT-CD8+ counts.

## METHOD

### Design of Study

Interim analysis of data from a randomized, parallel, controlled, single-blind clinical trial conducted to verify the efficacy of ILIB in reducing VL and increasing LT-CD4+ and LT-CD8+ counts in PLWHA. Interim analysis consists of comparisons among intervention groups performed before the formal conclusion of the study, usually before recruitment ends. It is often used in conjunction with stopping rules; thus, a study can be stopped if participants are exposed to unnecessary risk^(18)^. This study was registered in the Brazilian Clinical Trials Registry (ReBEC: RBR-7j9rv5d – U1111-1262-3868) on January 27, 2021. Data collection took place from February 2021 to December 2022.

### Local

The study was carried out in a Specialized Outpatient Service (*SAE*) of Infectology in an inland city of the State of São Paulo. The Infectology *SAE* is a public service that is part of the Brazilian Public Health System (*SUS*), which provides multidisciplinary assistance to PLWHA, chronic hepatitis B and C, individuals with HTLV-I/II infection, and individuals who are victims of accidents (occupational or sexual) with a biological risk of contracting infections by the aforementioned etiological agents. During the study period, the service monitored approximately 1,000 PLWHA.

### Population

All PLWHA who were undergoing outpatient follow-up and had detectable VL and were on the same ART treatment regimen were invited to participate in the study.

### Selection Criteria

PLWHA of both sexes, aged ≥ 18 years, undergoing outpatient treatment, with detectable VL and treated with tenofovir + lamivudine + dolutegravir (regardless of treatment duration) were included. Pregnant women, people with glaucoma, institutionalized people, and those who were unable to complete the form due to cognitive impairment were excluded.

### Sample Definition and Interim Analysis

Considering the clinical hypothesis that there is an improvement greater than 0.5 log of viral load in the treated group, given up to 5% of dropout, alpha of 5% (one-tailed) and power of 80%, the sample was estimated at 86 participants (43 participants in each treatment group).

Due to the absence of other studies on ILIB that presented references for viral load reduction and increase in LT-CD4+ and LT-CD8+ in PLWHA, it was decided that an interim analysis would be performed after 28 treatments. At that time, the study was interrupted (due to futility), as no mean of 0.5 log higher viral load was found in the intervention group.

### Data Collection

The present study included 28 participants, of which 24 attended the outpatient clinic and four received home visits according to the inclusion and exclusion criteria.

The researchers involved have training courses in laser therapy and have undergone training to standardize the application of the laser in the study. The main researcher (nurse) carried out nursing consultations for both groups that participated in the research. On the first day, a sociodemographic questionnaire was administered and participants were asked about the time of diagnosis, current or past opportunistic disease, other medications used, and the number of times they forgot to take ART in a month. A questionnaire was also administered on the first and last day of sessions to assess the incidence of nausea, vomiting, weakness, sweating, insomnia, lack of appetite, diarrhea, or other symptoms.

All participants collected the tests (viral load, LT-CD4+ and LT-CD8+) 10 days before starting treatment, as this is the time for the results to be available in the Laboratory Test Control System (Siscel) of the National Network.

All samples were collected by venipuncture from the participants’ upper limbs and stored in tubes with ethylenediaminetetraacetic acid (EDTA). The samples were processed on the Abbott M2000SP/M2000RT equipment with extraction, amplification and detection of the material in the laboratory of the referenced hospital of the teaching institution.

The selected participants were randomized into two groups: ILIB group and control group (CTRL). The randomization of the blocks was performed by an external person and the randomization sequence through a computer software.

As this was a single-blind study, participants were not informed about the treatment they received. Data analysis was performed by a third person who was unaware of group allocations.

In a comfortable environment, participants were invited to sit on a chair or lie down on a stretcher, according to their preference. Blood pressure, heart rate, and oximetry were measured before and at the end of all sessions, as a safety parameter for the systemic effects of light. No significant changes were observed.

Both participants and researchers wore protective eyewear. The researchers palpated the radial pulse and a bracelet with an attached device was placed on the wrist.

The device was calibrated and set by the supporting company for only two functions: control and intervention. For the intervention function, the red light was activated, with sound emission and a 30-minute timer displayed on the device. For the control function, the light was not activated, with only the sound emission and the timer being displayed on the device.

The ILIB group received treatment with *laser* for 30 minutes daily for 10 days, except Saturdays, Sundays and holidays. After the first cycle, participants rested for 20 days; then treatment was continued for another 10 days. The CTRL group was subjected to the same regimen, but using the radiation technique without laser. The protocol used was adapted from the traditional ILIB protocol for systemic treatments, available in the application of the company supplying the device (DMC Protocols). In the original protocol, a new 20-day break is recommended, followed by another 10 days of sessions. However, we chose to follow a previous study that applied an adapted protocol and obtained positive results^(13)^. All participants used the same protocol for the study. The sessions were standardized for the morning period, with prior scheduling, being held between 8 am and 10 am for all participants.

At the end of the last 10 days of ILIB, samples were collected from both groups for VL, LT-CD4+ and LT-CD8+ testing; the questionnaire used to assess the complaints and differences in perspective of the participants from the beginning to the end of the treatment period was reapplied. This questionnaire was self-administered or administered by a third person who was not part of the study to avoid response bias.

All participants completed 20 ILIB sessions. The irradiation parameters used in this study were: Equipment: Therapy Ilib, DMC Equipamentos®; Light source: laser (diode); Wavelength: 660nm; Power: 100 mW; Operating mode: continuous; Area of *“spot”*: 0.0984 cm^2^; Irradiation time: 30 minutes; Total energy: 180J; Energy density: 1,830 J/cm^2^ or 1.83 KJcm^2^; Irradiation site: wrist; Blood vessel: radial artery; Coupling: skin contact; Session frequency: 10 continuous days, 20-day interval, and then another 10 days.

All materials used (equipment, wristband, protective glasses, and cushion) were sanitized at the end of each session and between each participant, using cotton and 70% alcohol, as recommended by the equipment manufacturer.

### Data Analysis

The variables were stored in an Excel spreadsheet and analyzed descriptively through the *software* from the *International Business Machines Corporation* (IBM) – *Statistical Package for the Social Sciences* (SPSS) 25.0.

Chi-square trend tests were used to categorize the variables, whose occurrences were evaluated in each group and at each time point.

The Mann-Whitney U test was used to compare baseline data between the two groups, and a generalized linear mixed- effects model with gamma regression was used for longitudinal comparison between the two groups and to estimate effect sizes.

### Ethical Aspects

This study was approved (Opinion: 4,419,222) by the Research Ethics Committee (CEP) of the Medical School of Botucatu and the procedures were carried out following all ethical standards according to Resolution No. 466, of December 12, 2012^(19)^. All participants signed the Free and Informed Consent Form before starting the study.

The device used during the study was donated and the supporting company did not interfere at any time with the results of this research.

## RESULTS

**Figure 1:**
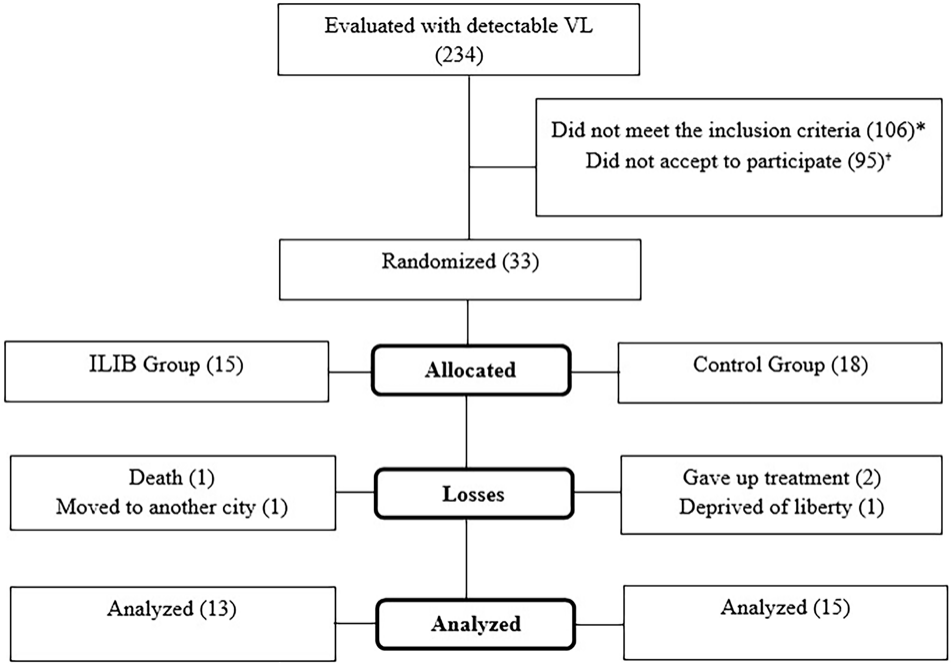
CONSORT flowchart of the research sample inclusion, allocation, follow-up, and analysis procedures.

A total of 28 participants were recruited (ILIB group: 13 and CTRL group: 15). The flowchart according to *Consolidated Standards of Reporting Trials* (CONSORT)^(20)^ is shown in Figure 1.

* Pregnant women, people with glaucoma, institutionalized people or people with cognitive impairment; ^†^ = Due to work or lack of transportation.

The services were carried out at an Infectious Diseases SAE linked to the *SUS*. All participants were exclusive users of the public health system, which can be attributed to the fact that PLWHA with access to health plans tend to seek care in private services. This aspect helps to explain the sociodemographic profile observed in the sample, characterized by low education and low income. Demographic data are presented in Table 1.

**Table 1:** Distribution of patients according to sociodemographic characteristics – Botucatu, SP, Brazil, 2021–2022.

Variables	ILIB Group n (%)	CTRL Group n (%)	Total n (%)
Sex			
Male	9 (32.1)	11 (39.3)	20 (71.4)
Female	4 (14.3)	4 (14.3)	8 (28.6)
Age	46 (24–64)*	38.2 (18–64)*	42 (18–64)*
Color			
White	11 (39.3)	10 (35.7)	24 (75)
Brown	2 (7.1)	3 (10.7)	5 (17.9)
Black	0 (0)	2 (7.1)	2 (7.1)
Marital status			
Single	8 (28.6)	11 (39.3)	19 (67.9)
Married	2 (7.1)	12 (7.1)	4 (14.3)
Divorced	2 (7.1)	1 (3.6)	3 (10.7)
Living together	1 (3.6)	1 (3.6)	2 (7.1)
Level of Education			
Unfinished elementary school	4 (14.3)	7 (25)	11 (39.3)
Unfinished high school	5 (17.9)	5 (17.9)	10 (35.7)
Finished high school	1 (3.6)	3 (10.7)	4 (14.3)
Higher Education	3 (10.7)	0 (0)	3 (10.7)
Family Income (Per Capita)	R$ 1,491.67 (275.00–5,000)*	R$803.21 (166.66–2,500.00)*	R$1,122.85 (166.66–5,000)*
Origin			
DRS** – Bauru			28 (100)

*Mean (Minimum – Maximum)

**DRS – Regional Health Department

Table 2 shows the complaints that participants presented on the first and last day of treatment. There was an improvement in all reported complaints, but not statistically significant.

**Table 2:** Initial and final questionnaire regarding complaints (n = 28) – Botucatu, SP, Brazil, 2021–2022.

Complaints	Before n (%)	After n (%)
Nausea		
Yes	5 (17.8)	1 (3.6)
Vomiting		
Yes	1 (3.6)	0 (0)
Weakness		
Yes	15 (53.6)	5 (17.9)
Sweating		
Yes	5 (17.9)	1 (3.6)
Insomnia		
Yes	13 (46.4)	2 (7.1)
Loss of appetite		
Yes	6 (21.4)	2 (7.1)
Diarrhea		
Yes	3 (10.4)	1 (3.6)

There were no differences in the baseline characteristics of the participants. Table 3 presents the 20 sessions before and after the interventions. There were no differences between the groups regarding LT-CD4+, LT-CD8+ and VL after the interventions (p > 0.17). A non-significant reduction in LT-CD8+ values was observed in the ILIB group at the end of treatment. Both groups reduced VL, with 40% of participants in the CTRL group and 61.5% in the ILIB group achieving undetectable VL at the end of treatment (p = 0.449).

**Table 3:** LT-CD4+, LT-CD8+ and VL values before and after treatment (n = 28) – Botucatu, SP, Brazil, 2021–2022.

	Team	Group		Diff (95% CI)^‡^	p-value
		ILIB* $\bar{x}$ (*s*)	CTRL $\bar{x}$ (*s*)		
LT-CD4+	D0	346 (204)	452 (353)	−7 (−199–186)	0.944
	D20	403 (239)	520 (338)	−117 (−336–102)	0.287
	Diff (95% CI)	57 (−129–242)	181 (−44–406)		
LT-CD8+	D0	981 (667)	838 (374)	55 (−330–440)	0.775
	D20	918 (361)	1,119 (337)	−197 (−483–89)	0.173
	Diff (95% CI)	74 (−278–424)	215 (−111–542)		
CV^§^(Log)	D0	2.8 (1.2)	3.7 (1.3)	−0.8 (−1.8–0.1)	0.086
	D20	1.1 (1.4)	1.4 (1.5)	−0.3 (−1.4–0.8)	0.597
	Diff (95% CI)	−1.7 (−2.8– −0.7)	−2.3 (−3.3– −1.3)		

*ILIB = *intravenous laser irradiation of blood*

^†^CTRL = control

^‡^Diff (CI 95%) = Difference and confidence interval between D0 to D20, or ILIB to CTRL

^§^VL = viral load

Table 4 shows the individual evaluation related to the increase or decrease in LT-CD8+ values and undetectable viral load at the end of the protocol. It was observed that of the seven patients who presented a drop in LT-CD8+ in the ILIB group, five had an undetectable viral load and, of these five, three had opportunistic disease during treatment.

**Table 4:** Distribution of study participants in relation to LT+CD8+, undetectable viral load and opportunistic disease (n = 28) – Botucatu, SP, Brazil, 2021–2022.

	n	LT-CD8+	
		Undetectable CV*	Opportunistic disease
**CTRL^†^**	**15**	**6 (40%)**	–
Increased LT-CD8+	11	4	
LT-CD8+ reduction	4	2	
**ILIB^‡^**	**13**	**8 (61.5%)**	
Increased LT-CD8+	6	3	
LT-CD8+ reduction	7	5	3
**Grand total**	**28**		

*VL = viral load

^†^CTRL = control

^‡^ILIB = *intravenous laser irradiation of blood*

Given the results found, and the impossibility of continuing with the treatment protocol, the study was prematurely interrupted during the interim analysis.

## DISCUSSION

To date, this was the first randomized, parallel, controlled, single-blind clinical trial that verified the efficacy of ILIB, used together with ART, in reducing VL and increasing LT-CD4+ and LT-CD8+ counts in PLWHA. After 20 sessions, there were no significant differences in LT-CD4+, LT-CD8+, and VL changes between the ILIB and CTRL groups.

In our study, it was observed that the participants in the ILIB group who presented a reduction in the LT-CD8+ count, for the most part, had an undetectable viral load (VL). However, they also developed opportunistic diseases during treatment, which may explain the decrease in LT-CD8+. Previous studies suggest that factors such as advanced age, low LT-CD4+ counts at the start of antiretroviral therapy, or the use of injectable drugs may be associated with inadequate immune response^(21–23)^. Additionally, vaccines can temporarily interfere with LT-CD4+ levels, which tend to normalize between two and six weeks after administration^(24)^.

VL is expected to be undetectable within six months of starting ART^(25)^. In the present study, the majority of participants in both groups had undetectable VL at the end of treatment.

Some people do not achieve undetectable VL, which may be due to lack of adherence to treatment or virological failure^(3)^. One of the reasons for lack of adherence to treatment is the occurrence of adverse reactions related to ART, such as nausea, vomiting, headache, insomnia, among others^(26)^. In the present study, PLWHA reported improvement in general complaints after ILIB therapy.

Improvement in weakness and sleep was also evident in the CTRL group. These results observed in the untreated group can be attributed to the participant’s expectations, treatment experience, and the fact that they received care^(27)^. Many factors, such as quality of care, memory, the location where treatment was performed, and patient-provider interaction^(28,29,30)^ can affect treatment outcomes.

The Society for Interdisciplinary Placebo Studies defined placebo “effects” as specific changes resulting from the placebo regimen, such as neurobiological and psychological expectancy mechanisms; however, “response” to placebo was defined as changes in the patient’s health as a result of placebo use, with regression of the natural course of the disease^(31)^.

This study has some limitations. First, difficulties were encountered in recruiting eligible participants due to the adopted protocol, which consisted of 10 sessions held on consecutive days, followed by a 20-day break, and then another 10 consecutive sessions, making it impossible for many PLWHA to participate. It is worth remembering that the per capita income of the participants was 1,122.85 reais, which highlights the social vulnerability of the group.

Although this protocol is recommended by the company supplying the device to act on the immune system, it proved to be unfeasible for the context of the present study. In contrast, research using ILIB with a frequency of two weekly sessions reported positive effects^(12–32)^, which may represent a viable alternative to improve adherence and increase the number of participants in a future study. It should be noted that a standardized protocol based on the participants’ body type was not adopted. Furthermore, the literature still lacks specific recommendations that relate body biotype to the definition of application time and dosimetry, with existing studies being inconclusive^(33,34,35)^.

Second, the number of individuals with detectable VL in the sample was relatively small, as most people start ART soon after diagnosis and have undetectable VL. Furthermore, participants with detectable VL often already had low adherence to ART, a factor that also negatively impacted adherence to the study protocol.

Finally, another limitation was that participants were not asked, in all sessions, about the presence of symptoms indicative of infection or recent vaccination history. These factors may have contributed to the reduction in LT-CD4+ and LT-CD8+ counts observed in some cases, being a possible confounding variable in the results. However, it was also not assessed whether participants of biological female sex were in different phases of the menstrual cycle during treatment. A study indicates that hormonal fluctuations throughout the cycle can modulate immunity, with evidence of a transient reduction in T-lymphocyte counts during menstruation, especially in HIV-positive women, which may influence the immune response and the interpretation of laboratory parameters^(36)^.

On the other hand, as contributions of the present study, protocols applicable in clinical contexts are suggested, with the adoption of approaches with a smaller number of sessions and more detailed clinical evaluations of participants regarding any symptoms of infections, vaccination status, or lack of immune response to justify better control of LT-CD4+ and LT-CD8+ counts.

## CONCLUSION

There were no differences in LT-CD4+, LT-CD8+, and viral load values in PLWHA who received ILIB as adjuvant treatment to ART.

However, treated participants who experienced a reduction in CD8+ count had undetectable VL.

Considering the scarcity of clinical studies evaluating PLWHA undergoing ILIB, it is understood that conducting other studies with protocols applicable in clinical contexts with a smaller number of sessions, more detailed clinical evaluations of participants, and a larger sample can yield significant results.

## Data Availability

The entire dataset supporting the findings of this study is available upon request from the corresponding author (Fabiana Tomé Ramos). The dataset is not publicly available as it contains information that could compromise the privacy of research participants.
