# Supplementary material for: Effects of intravenous laser irradiation on viral load and T lymphocytes in people living with HIV/AIDS
Source: Rev Esc Enferm USP. 2025 Sep 5;59:e20250004. doi: 10.1590/1980-220X-REEUSP-2025-0004en (PMC12416369; doi:10.1590/1980-220X-REEUSP-2025-0004en)
Supplement: Translation

# Translation: Efeitos da irradiação intravascular com *laser* na carga viral e linfócitos T em pessoas vivendo com HIV/aids

**DOI:** 10.1590/1980-220X-REEUSP-2025-0004pt

**Authors:** Fabiana Tomé Ramos, Maria Cecília Yoshioka Lobo, Hélio Amante Miot, Eliana Maria Minicucci, Rúbia Aguiar Alencar

**Affiliations:** 1 Universidade Estadual Paulista, Faculdade de Medicina, Botucatu, SP, Brasil.

**Keywords:** Terapia com Luz de Baixa Intensidade, HIV, Síndrome de Imunodeficiência Adquirida, Contagem de Linfócitos, Carga Viral

## Abstract

**Objetivo:**

Avaliar a eficácia da irradiação intravascular de sangue com *laser* na redução da carga viral e aumento de LT-CD4+ e LT-CD8+ em pessoas vivendo com HIV/aids.

**Método:**

Ensaio clínico randomizado, controlado, paralelo e uni-cego. Vinte e oito participantes foram alocados nos grupos intervenção (ILIB *n*= 15) e controle (CTRL *n*= 13). O grupo ILIB recebeu irradiação com *laser* vermelho (660nm), aplicado sobre a artéria radial (30 minutos/dia por 20 dias), com energia total de 180 J. O grupo CTRL recebeu intervenção simulada (sem emissão de luz). Carga viral e contagens de LT-CD4+ e LT-CD8+ foram avaliadas ao final.

**Resultados:**

Ambos os grupos apresentaram redução da carga viral (ILIB: −1,7 log; CTRL: −2,3 log). No grupo ILIB, 61,5% dos participantes atingiram carga viral indetectável (p = 0,449). Não houve aumento na contagem de LT CD4+ (p = 0,287), mas uma redução não significativa na contagem de LT CD8+ foi observada no grupo ILIB (p = 0,173). Não houve diferenças estatisticamente significativas entre os grupos para nenhum desfecho (p > 0,17). O estudo foi interrompido prematuramente durante a análise interina.

**Conclusão:**

A ILIB, como terapia adjuvante à TARV, não apresentou superioridade clínica ou estatística nos parâmetros imunológicos e virológicos avaliados.

**Registro de Ensaio (ReBEC):**

RBR-7j9rv5d (https://ensaiosclinicos.gov.br/trial/10848)

## INTRODUÇÃO

Embora existam terapias promissoras associadas à cura do HIV, não há evidências suficientes para afirmar isso. Ainda não existe cura para a infecção pelo HIV, mas a atual terapia antirretroviral (TARV) é eficaz. O objetivo do tratamento é inibir a replicação do vírus no organismo; preservar/restaurar o sistema imunológico; reduzir a probabilidade de surgimento de cepas virais mais resistentes; diminuir as taxas de morbidade, mortalidade e hospitalização; diminuir o risco de desenvolver doenças oportunistas e aumentar a duração e a qualidade de vida das pessoas que vivem com HIV/síndrome da imunodeficiência adquirida (AIDS) (PVHA)^(1)^. Este tratamento tem um grande impacto na sobrevida das PVHA, alterando assim a caracterização epidemiológica do HIV/aids^(1)^.

O Protocolo Clínico e Diretrizes Terapêuticas para o Manejo da Infecção pelo HIV em Adultos do Ministério da Saúde afirma que o objetivo do acompanhamento e tratamento é atingir uma carga viral (CV) indetectável^(2)^. Porém, mesmo com a TARV e o acompanhamento, algumas PVHA não atingem esse nível, o que pode ocorrer devido à má adesão ao tratamento e à resistência viral^(3)^.

Uma estratégia sugerida para alcançar uma CV indetectável em PVHA é a utilização da irradiação intravascular de sangue com *laser,* do inglês *intravenous laser irradiation of blood* (ILIB), como alternativa terapêutica adjuvante.

Os *lasers*, desde sua criação, passaram por grande evolução tecnológica nas áreas da física e da medicina, sendo aplicados no tratamento de diversas patologias. Eles consistem em um meio ativo—sólido, líquido ou gasoso—que emite luz ao ser excitado por uma fonte de energia^(4)^. A introdução da terapia a *laser* de baixa potência, em 1966, consolidou-se como a modalidade terapêutica mais investigada no campo das aplicações clínicas do *laser* ^(4)^.

Originalmente, a ILIB era realizada por meio de punção venosa na qual o dispositivo era acoplado. Atualmente, com o avanço tecnológico, pode ser feita de forma transcutânea, próxima aos vasos sanguíneos, ou seja, em contato com a pele, sem necessidade de punção^(5)^. A partir disso, em 2021 foi sugerido que o termo utilizado para descrever este procedimento não invasivo fosse “fotobiomodulação vascular”^(6)^. No entanto, ainda há divergência na literatura quanto à terminologia utilizada, com estudos referenciando como “ILIB transcutâneo”^(7)^, “ILIB modificado”^(8)^, “fotobiomodulação sistêmica vascular”^(9)^, entre outros. Assim, no presente estudo, considerando-se que o termo ILIB continua a ser utilizado de maneira frequente, optamos por denominar a técnica como ILIB, de maneira mais generalizada.

A ação do ILIB atua ao modular a resposta imune, estimulando diretamente enzimas antioxidantes, como a superóxido dismutase (SOD), catalase e glutationa peroxidase^(10)^. Essas enzimas têm a função de neutralizar os radicais livres presentes no organismo, reduzindo o estresse oxidativo, que é um fator comum em diversas condições inflamatórias e infecciosas^(10)^.

Em pessoas vivendo com HIV, o estresse oxidativo está intensificado pela presença contínua do vírus, podendo contribuir para a progressão da doença e para o surgimento de comorbidades^(11)^. Com isso, o ILIB pode atuar como terapia adjuvante ao modular o sistema imune e reduzir o estresse oxidativo. Esse efeito sistêmico pode favorecer a preservação das células T CD4+ e melhorar a resposta imune, contribuindo para a redução da progressão viral e de comorbidades associadas.

Estudos clínicos mostram efeitos positivos da ILIB na regulação da pressão arterial, glicose, triglicerídeos e colesterol^(12)^, melhora da trombocitopenia, neutropenia e distúrbios de hemoglobina em pacientes quimioterápicos^(13)^, proteção contra o envelhecimento ovariano, melhora da independência de pacientes pós-AVC^(14)^, melhora de sintomas em pessoas com COVID-19 prolongada^(15)^, dor e distúrbios do sono^(16)^, entre outros.

Contudo, somente um estudo foi encontrado com o uso da ILIB em PVHA. Nesse estudo, a ILIB foi utilizada nas regiões dos vasos cubitais, fígado e timo, apresentando redução nos sinais e sintomas de PVHA e hepatite, aumento nas contagens de linfócitos T-CD4+ (LT-CD4+), linfócitos T-CD8+ (LT-CD8+) e linfócitos totais e diminuição da CV em participantes com infecção pelo HIV^(17)^. Esse estudo foi realizado a partir de 1995 em pessoas que ainda não usavam TARV. E, após 1998, a ILIB foi introduzida como tratamento para algumas complicações da TARV, como náuseas, intoxicação e comprometimento do estado geral, apresentando efeitos positivos e aumento da contagem de leucócitos^(17)^.

Os autores do estudo acreditaram que a irradiação do *laser* poderia interferir no ciclo celular alterado pela infecção, levando ao aumento da microcirculação sanguínea e à regeneração das células danificadas. Observou-se também que a carga viral plasmática permaneceu baixa por muito tempo após o término do tratamento com o ILIB^(17)^.

Diante da escassez de estudos sobre ILIB em PVHA, o presente estudo teve como objetivo avaliar a eficácia da ILIB na redução da CV e no aumento da contagem de LT-CD4+ e LT-CD8+ em PVHA. A hipótese do estudo foi que PVHA submetidas a ILIB apresentariam redução da CV e aumento da contagem de LT-CD4+ e LT-CD8+.

## MÉTODO

### Tipo de Estudo

Análise interina dos dados de um ensaio clínico randomizado, paralelo, controlado e uni-cego conduzido para verificar a eficácia da ILIB na redução da CV e no aumento da contagem de LT-CD4+ e LT-CD8+ em PVHA. A análise interina consiste em comparações entre grupos de intervenção realizadas antes da conclusão formal do estudo, geralmente antes do término do recrutamento. É frequentemente utilizada em conjunto com regras de interrupção; assim, um estudo pode ser interrompido se participantes forem expostos a risco desnecessário^(18)^. Este estudo foi registrado no Registro Brasileiro de Ensaios Clínicos (ReBEC: RBR-7j9rv5d – U1111-1262-3868) em 27 de janeiro de 2021. A coleta de dados aconteceu de fevereiro de 2021 a dezembro de 2022.

### Local do Estudo

O estudo foi realizado em um Serviço de Ambulatórios Especializados (SAE) de Infectologia no interior do Estado de São Paulo. O SAE de Infectologia é um serviço público integrante do Sistema Único de Saúde (SUS), que oferece assistência multidisciplinar às PVHA, hepatites crônicas por vírus B e C, indivíduos com infecção pelo HTLV – I/II, indivíduos vítimas de acidentes (ocupacionais ou sexuais) com risco biológico de contrair infecções pelos agentes etiológicos citados. No período do estudo, o serviço acompanhava cerca de 1.000 PVHA.

### População

Foram convidadas a participar da pesquisa todas as PVHA que faziam acompanhamento ambulatorial e que estavam com CV detectável e em mesmo esquema terapêutico da TARV.

### Critérios de Seleção

Foram incluídas PVHA de ambos os sexos, com idade ≥ 18 anos, em tratamento ambulatorial, com CV detectável e tratadas com tenofovir + lamivudina + dolutegravir (independentemente do tempo de tratamento). Foram excluídas gestantes, pessoas com glaucoma, pessoas institucionalizadas e aqueles que não conseguiram preencher o formulário por comprometimento cognitivo.

### Definição da Amostra e Análise Interina

Considerando a hipótese clínica de que haja melhora superior a 0,5 log da carga viral no grupo tratado, dado até 5% de *dropout*, alfa de 5% (unicaudal) e poder de 80%, a amostra foi estimada em 86 participantes (43 participantes em cada grupo de tratamento).

Devido à ausência de outros estudos sobre ILIB que apresentassem referências para redução da carga viral e aumento de LT-CD4+ e LT-CD8+ em PVHA, optou-se por realizar uma análise interina que foi realizada após 28 tratamentos. Nesse momento, o estudo foi interrompido (por futilidade), pois não foi identificada média de 0,5 log da carga viral superior no grupo de intervenção.

### Coleta de Dados

O presente estudo incluiu 28 participantes, sendo que 24 compareceram ao ambulatório e quatro receberam visitas domiciliares de acordo com os critérios de inclusão e exclusão.

Os pesquisadores envolvidos possuem cursos de formação em laserterapia e passaram por treinamento para a padronização da aplicação do *laser* no estudo. A pesquisadora principal (enfermeira) realizou consulta de enfermagem para ambos os grupos que participaram da pesquisa. No primeiro dia foi aplicado um questionário sociodemográfico e foram questionados sobre o tempo de diagnóstico, doença oportunista presente ou passada, outros medicamentos utilizados e o número de vezes que se esqueceram de tomar a TARV em um mês. Também foi aplicado questionário no primeiro e no último dia de sessões para avaliar a incidência de náuseas, vômitos, fraqueza, sudorese, insônia, falta de apetite, diarreia ou outros sintomas.

Todos os participantes coletaram os exames (carga viral, LT-CD4+ e LT-CD8+) 10 dias antes do início do tratamento, pois é o tempo para que o resultado esteja disponível no Sistema de Controle de Exames Laboratoriais (Siscel) da Rede Nacional.

Todas as amostras foram coletadas por punção venosa dos membros superiores dos participantes e armazenadas em tubos com ácido etilenodiamino tetraacético (EDTA). As amostras foram processadas no equipamento Abbott M2000SP/M2000RT com extração, amplificação e detecção do material no laboratório do hospital referenciado da instituição de ensino.

Os participantes selecionados foram randomizados em dois grupos: grupo ILIB e grupo controle (CTRL). A randomização dos blocos foi realizada por pessoa externa e a sequência de randomização foi realizada por meio de *software* computacional.

Como se tratava de um estudo uni-cego, os participantes não foram informados sobre o tratamento que receberam. A análise dos dados foi realizada por uma terceira pessoa que desconhecia as alocações dos grupos.

Em ambiente confortável, os participantes foram convidados a sentar-se em cadeira ou se deitarem em maca, conforme sua preferência. A pressão arterial, frequência cardíaca e oximetria foram aferidas antes e ao final de todas as sessões, como parâmetro de segurança para os efeitos sistêmicos da luz. Não foram observadas alterações significativas.

Tanto os participantes quanto os pesquisadores usaram óculos de proteção. Os pesquisadores apalparam o pulso radial e uma pulseira com um dispositivo acoplado foi colocada no pulso.

O aparelho foi calibrado e configurado pela empresa apoiadora para apenas duas funções: controle e intervenção. Para a função de intervenção foi acionada a luz vermelha, com emissão de som e cronômetro de 30 minutos apresentado no *display* do aparelho. Para a função de controle, a luz não foi acionada, sendo exibidos no *display*do aparelho apenas a emissão sonora e o cronômetro.

O grupo ILIB recebeu tratamento com *laser* por 30 minutos diários durante 10 dias, exceto sábados, domingos e feriados. Após o primeiro ciclo, os participantes descansaram por 20 dias; então, o tratamento foi continuado por mais 10 dias. O grupo CTRL foi submetido ao mesmo regime, mas utilizando a técnica de radiação sem *laser*. O protocolo utilizado foi adaptado do protocolo ILIB tradicional para tratamentos sistêmicos, disponível no aplicativo da empresa fornecedora do dispositivo (DMC Protocolos). No protocolo original, recomenda-se uma nova pausa de 20 dias, seguida por mais 10 dias de sessões. No entanto, optamos por seguir um estudo anterior que aplicou um protocolo adaptado e obteve resultados positivos^(13)^. Todos os participantes utilizaram o mesmo protocolo para o estudo. As sessões foram padronizadas para o período da manhã, com agendamento prévio, sendo realizadas entre 8h e 10h para todos os participantes.

Ao final dos últimos 10 dias da ILIB, foram coletadas amostras de ambos os grupos para testes de CV, LT-CD4+ e LT-CD8+; o questionário utilizado para avaliar as queixas e diferenças de perspectiva dos participantes desde o início até o final do período de tratamento foi reaplicado. Este questionário foi autoaplicado ou administrado por uma terceira pessoa que não fez parte do estudo para evitar viés de resposta.

Todos os participantes completaram 20 sessões de ILIB. Os parâmetros de irradiação utilizados neste estudo foram: Equipamento: Therapy Ilib, DMC Equipamentos®; Fonte de luz: *laser* (diodo); Comprimento de onda: 660nm; Potência: 100 mW; Modo de operação: contínuo; Área do *“spot”*: 0,0984 cm^2^; Tempo de irradiação: 30 minutos; Energia total: 180 J; Densidade energética: 1.830 J/cm^2^ ou 1,83 KJ cm^2^; Local de irradiação: pulso; Vaso sanguíneo: artéria radial; Acoplamento: contato com a pele; Frequência da sessão: 10 dias contínuos, intervalo de 20 dias e depois mais 10 dias.

Todos os materiais utilizados (equipamento, pulseira, óculos de proteção e almofada) foram higienizados no final de cada sessão e entre cada participante, utilizando algodão e álcool 70%, conforme recomendado pelo fabricante do equipamento.

### Análise de Dados

As variáveis foram armazenadas em planilha *Excel* e analisadas descritivamente por meio do *software* da *International Business Machines Corporation* (IBM) – *Statistical Package for the Social Sciences* (SPSS) 25.0.

Foram utilizados testes de tendência qui-quadrado para categorização das variáveis, cujas ocorrências foram avaliadas em cada grupo e em cada momento.

O teste *U* de *Mann-Whitney* foi utilizado para comparar os dados iniciais entre os dois grupos, e um modelo linear generalizado de efeitos mistos com regressão gama foi utilizado para comparação longitudinal entre os dois grupos e para estimar os tamanhos dos efeitos.

### Aspectos Éticos

O presente estudo foi aprovado (Parecer: 4.419.222) pelo Comitê de Ética em Pesquisa (CEP) da Faculdade de Medicina de Botucatu e os procedimentos foram realizados seguindo todas as normas éticas conforme Resolução nº 466, de 12 de dezembro de 2012^(19)^. Todos os participantes assinaram o Termo de Consentimento Livre e Esclarecido antes do início da participação na pesquisa.

O dispositivo utilizado durante o estudo foi doado e a empresa apoiadora não interferiu em nenhum momento nos resultados desta pesquisa.

## RESULTADOS

Um total de 28 participantes foi recrutado (grupo ILIB: 13 e grupo CTRL: 15). O fluxograma de acordo com *Consolidated Standards of Reporting Trials* (CONSORT)^(20)^ é mostrado na Figura 1.

**Figura 1:** Fluxograma CONSORT dos procedimentos de inclusão, alocação, seguimento e análise da amostra da pesquisa.

* Gestantes, pessoas com glaucoma, pessoas institucionalizadas ou com déficit cognitivo; ^†^ = Devido ao trabalho ou falta de transporte.

Os atendimentos foram realizados em um SAE de Infectologia vinculado ao SUS. A totalidade dos participantes era usuária exclusiva do sistema público de saúde, o que pode ser atribuído ao fato de que PVHA com acesso a planos de saúde tendem a buscar atendimento em serviços privados. Esse aspecto ajuda a explicar o perfil sociodemográfico observado na amostra, caracterizado por baixa escolaridade e baixa renda. Os dados demográficos são apresentados na Tabela 1.

**Tabela 1:** Distribuição dos pacientes segundo características sociodemográficas – Botucatu, SP, Brasil, 2021–2022.

Variáveis	Grupo ILIB n (%)	Grupo CTRL n (%)	Total – n (%)
Sexo			
Masculino	9 (32,1)	11 (39,3)	20 (71,4)
Feminino	4 (14,3)	4 (14,3)	8 (28,6)
Idade	46 (24–64)*	38,2 (18–64)*	42 (18–64)*
Cor			
Branca	11 (39,3)	10 (35,7)	24 (75)
Parda	2 (7,1)	3 (10,7)	5 (17,9)
Negra	0 (0)	2 (7,1)	2 (7,1)
Estado Civil			
Solteiro	8 (28,6)	11 (39,3)	19 (67,9)
Casado	2 (7,1)	12 (7,1)	4 (14,3)
Divorciado	2 (7,1)	1 (3,6)	3 (10,7)
Amasiado	1 (3,6)	1 (3,6)	2 (7,1)
Escolaridade			
Ensino Fundamental Incompleto	4 (14,3)	7 (25)	11 (39,3)
Ensino Médio Incompleto	5 (17,9)	5 (17,9)	10 (35,7)
Ensino Médio Completo	1 (3,6)	3 (10,7)	4 (14,3)
Superior	3 (10,7)	0 (0)	3 (10,7)
Renda Familiar (Per capita)	R$ 1.491,67 (275,00–5.000)*	R$803,21 (166,66–2.500,00)*	R$1.122,85 (166,66–5.000)*
Procedência			
DRS** – Bauru			28 (100)

*Média (Mínimo – Máximo)

**DRS – Departamento Regional de Saúde

A Tabela 2 mostra as queixas que os participantes apresentaram no primeiro e no último dia de tratamento. Houve uma melhora em todas as queixas relatadas, mas não estatisticamente significativa.

**Tabela 2:** Questionário inicial e final referente às queixas (n = 28) – Botucatu, SP, Brasil, 2021–2022.

Queixas	Antes n (%)	Depois n (%)
Náusea		
Sim	5 (17,8)	1 (3,6)
Vômito		
Sim	1 (3,6)	0 (0)
Fraqueza		
Sim	15 (53,6)	5 (17,9)
Sudorese		
Sim	5 (17,9)	1 (3,6)
Insônia		
Sim	13 (46,4)	2 (7,1)
Inapetência		
Sim	6 (21,4)	2 (7,1)
Diarreia		
Sim	3 (10,4)	1 (3,6)

Não houve diferenças nas características basais dos participantes. A Tabela 3 apresenta as 20 sessões anteriores e posteriores das intervenções. Não houve diferenças entre os grupos em relação a LT-CD4+, LT-CD8+ e CV após as intervenções (p > 0,17). Observou-se redução não significativa nos valores de LT-CD8+ no grupo ILIB ao final do tratamento. Ambos os grupos reduziram a CV, sendo que 40% dos participantes do grupo CTRL e 61,5% do grupo ILIB atingiram CV indetectável ao final do tratamento (p = 0,449).

**Tabela 3:** Valores de LT-CD4+, LT-CD8+ e CV antes e depois do tratamento (n = 28) – Botucatu, SP, Brasil, 2021–2022.

	Time	Grupo		Dif (CI 95%)^‡^	p-valor
		ILIB* $\bar{x}$ (*s*)	CTRL $\bar{x}$ (*s*)		
LT-CD4+	D0	346 (204)	452 (353)	−7 (−199–186)	0.944
	D20	403 (239)	520 (338)	−117 (−336–102)	0.287
	Dif (CI 95%)	57 (−129–242)	181 (−44–406)		
LT-CD8+	D0	981 (667)	838 (374)	55 (−330–440)	0.775
	D20	918 (361)	1,119 (337)	−197 (−483–89)	0.173
	Dif (CI 95%)	74 (−278–424)	215 (−111–542)		
CV^§^(Log)	D0	2.8 (1.2)	3.7 (1.3)	−0.8 (−1.8–0.1)	0.086
	D20	1.1 (1.4)	1.4 (1.5)	−0.3 (−1.4–0.8)	0.597
	Dif (CI 95%)	−1.7 (−2.8– −0.7)	−2.3 (−3.3– −1.3)		

*ILIB = *intravenous laser irradiation of blood*

^†^CTRL = controle

^‡^Dif (CI 95%) = Diferença e intervalo de confiança entre D0 para D20, ou ILIB para CTRL

^§^CV = carga viral

A Tabela 4 mostra a avaliação individual relacionada ao aumento ou diminuição dos valores de LT-CD8+ e com carga viral indetectável ao final do protocolo. Observou-se que dos sete pacientes que apresentaram queda de LT-CD8+ no grupo ILIB, cinco estavam com carga viral indetectável e, desses cinco, três estavam com doença oportunista durante o tratamento.

**Tabela 4:** Distribuição dos participantes do estudo em relação à LT+CD8+, carga viral indetectável e doença oportunista (n = 28) – Botucatu, SP, Brasil, 2021–2022.

		LT-CD8+	
	n	CV* indetectável	Doença oportunista
**CTRL^†^**	**15**	**6 (40%)**	–
Aumento LT-CD8+	11	4	
Redução LT-CD8+	4	2	
**ILIB^‡^**	**13**	**8 (61,5%)**	
Aumento LT-CD8+	6	3	
Redução LT-CD8+	7	5	3
**Total geral**	**28**		

*CV = carga viral;

^†^CTRL = controle

^‡^ILIB = *intravenous laser irradiation of blood*

Diante dos resultados encontrados, e a inviabilidade de continuar com o protocolo de tratamento, o estudo foi interrompido prematuramente durante a análise interina.

## DISCUSSÃO

Até o momento, este foi o primeiro ensaio clínico randomizado, paralelo, controlado e uni-cego que verificou a eficácia da ILIB, usada junto com a TARV, na redução da CV e no aumento da contagem de LT-CD4+ e LT-CD8+ em PVHA. Após 20 sessões, não houve diferenças significativas nas alterações de LT-CD4+, LT-CD8+ e CV entre os grupos ILIB e CTRL.

Em nosso estudo, observou-se que os participantes do grupo ILIB que apresentaram redução na contagem de LT-CD8+, em sua maioria, possuíam carga viral (CV) indetectável. No entanto, também desenvolveram doenças oportunistas durante o tratamento, o que pode justificar a diminuição dos LT-CD8+. Estudos anteriores sugerem que fatores como idade avançada, baixas contagens de LT-CD4+ no início da terapia antirretroviral ou o uso de drogas injetáveis podem estar associados à não resposta imunológica adequada^(21,22,23)^. Além disso, vacinas podem interferir temporariamente nos níveis de LT-CD4+, que tendem a se normalizar entre duas e seis semanas após a administração^(24)^.

Espera-se que a CV seja indetectável dentro de seis meses após o início da TARV^(25)^. No presente estudo, a maioria dos participantes de ambos os grupos apresentou CV indetectável ao final do tratamento.

Algumas pessoas não atingem uma CV indetectável, o que pode ser devido à falta de adesão ao tratamento ou à falha virológica^(3)^. Um dos motivos da falta de adesão ao tratamento é a ocorrência de reações adversas relacionadas à TARV, como náuseas, vômitos, cefaleia, insônia, entre outras^(26)^. No presente estudo, as PVHA relataram melhora das queixas gerais após a terapia com ILIB.

Também evidenciou-se no grupo CTRL, melhora da fraqueza e do sono. Esses resultados observados no grupo não tratado podem ser atribuídos à expectativa do participante, à experiência do tratamento e ao fato de terem recebido cuidados^(27)^. Muitos fatores podem afetar os resultados do tratamento, a qualidade do atendimento, a memória, o local onde o tratamento foi realizado e a interação paciente-profissional^(28,29,30)^.

A Sociedade para Estudos Interdisciplinares de Placebo *(Society for Interdisciplinary Placebo Studies)* definiu os “efeitos” do placebo como alterações específicas decorrentes do regime placebo, tais como mecanismos de expectativa neurobiológicos e psicológicos; entretanto, a “resposta” ao placebo foi definida como alterações na saúde do paciente como resultado do uso de placebo, com regressão do curso natural da doença^(31)^.

Este estudo apresenta algumas limitações. Primeiramente, enfrentaram-se dificuldades no recrutamento de participantes elegíveis devido ao protocolo adotado, que consistia em 10 sessões realizadas em dias consecutivos, seguidas por uma pausa de 20 dias, e posteriormente mais 10 sessões consecutivas, inviabilizando a participação de muitas PVHA. Vale lembrar que a renda per capita dos participantes foi de 1.122,85 reais, que evidencia a vulnerabilidade social do grupo.

Embora esse protocolo seja recomendado pela empresa fornecedora do dispositivo para atuação no sistema imunológico, mostrou-se inviável para a realidade do presente estudo. Por outro lado, pesquisas que empregaram a ILIB com frequência de duas sessões semanais relataram efeitos positivos^(12–32)^, o que pode representar uma alternativa viável para melhorar a adesão e aumentar o número de participantes num futuro estudo. Ressalta-se que não foi adotado um protocolo padronizado com base no biotipo corporal dos participantes. Além disso, a literatura ainda carece de recomendações específicas que relacionem biotipo corporal à definição do tempo de aplicação e da dosimetria, sendo os estudos existentes inconclusivos^(33,34,35)^.

Em segundo lugar, o número de indivíduos com CV detectável na amostra foi relativamente pequeno, visto que a maioria das pessoas inicia a TARV logo após o diagnóstico e apresenta CV indetectável. Além disso, os participantes com CV detectável frequentemente já apresentavam baixa adesão à TARV, fator que também impactou negativamente na adesão ao protocolo do estudo.

Por fim, outra limitação foi que os participantes não foram questionados, em todas as sessões, sobre a presença de sintomas indicativos de infecção ou histórico recente de vacinação. Esses fatores podem ter contribuído para a redução nas contagens de LT-CD4+ e LT-CD8+ observada em alguns casos, sendo uma possível variável de confusão nos resultados. Contudo, também não foi avaliado se as participantes do sexo biológico feminino estavam em diferentes fases do ciclo menstrual durante o tratamento. Estudo indica que as flutuações hormonais ao longo do ciclo podem modular a imunidade, com evidências de redução transitória na contagem de linfócitos T durante a menstruação, especialmente em mulheres HIV-positivas, o que pode influenciar a resposta imunológica e a interpretação de parâmetros laboratoriais^(36)^.

Por outro lado, como contribuições do presente estudo, sugerem-se protocolos aplicáveis em contextos clínicos, com a adoção de abordagens com menor número de sessões e avaliações clínicas mais detalhadas dos participantes quanto a quaisquer sintomas de infecções, estado vacinal ou falta de resposta imunológica para justificar o melhor controle das contagens de LT-CD4+ e LT-CD8+.

## CONCLUSÃO

Não houve diferenças nos valores de LT-CD4+, LT-CD8+ e carga viral em PVHA que receberam ILIB como tratamento adjuvante à TARV.

Entretanto, os participantes tratados que apresentaram redução na contagem de CD8+ apresentaram CV indetectável.

Considerando a escassez de estudos clínicos que avaliaram PVHA submetidas à ILIB, entende-se que a realização de outros estudos com protocolos aplicáveis em contextos clínicos com menor número de sessões, avaliações clínicas mais detalhadas dos participantes e maior amostra pode trazer resultados significativos.

## Data Availability

Todo o conjunto de dados que dá suporte aos resultados deste estudo está disponível mediante solicitação ao autor correspondente (Fabiana Tomé Ramos). O conjunto de dados não está publicamente disponível por conter informação que compromete a privacidade dos participantes da pesquisa.

# Translation: Efectos de la irradiación intravascular con *láser* en la carga viral y los linfocitos T en personas que viven con el VIH/sida

**DOI:** 10.1590/1980-220X-REEUSP-2025-0004es

**Authors:** Fabiana Tomé Ramos, Maria Cecília Yoshioka Lobo, Hélio Amante Miot, Eliana Maria Minicucci, Rúbia Aguiar Alencar

**Affiliations:** 1 Universidade Estadual Paulista, Faculdade de Medicina, Botucatu, SP, Brasil.

**Keywords:** Terapia por Luz de Baja Intensidad, VIH, Síndrome de Inmunodeficiencia Adquirida, Recuento de Linfocitos, Carga Viral

## Abstract

**Objetivo:**

Evaluar la eficacia de la irradiación intravascular de sangre con láser en la reducción de la carga viral y el aumento de LT-CD4+ y LT-CD8+ en personas viviendo con VIH/sida.

**Método:**

Ensayo clínico aleatorizado, controlado, paralelo y simple ciego. Se asignaron veintiocho participantes a los grupos de intervención (ILIB n = 15) y control (CTRL n = 13). El grupo ILIB recibió irradiación con láser rojo (660 nm), aplicado sobre la arteria radial (30 minutos/día durante 20 días), con una energía total de 180 J. El grupo CTRL recibió una intervención simulada (sin emisión de luz). Al final se evaluaron la carga viral y los recuentos de LT-CD4+ y LT-CD8+.

**Resultados:**

Ambos grupos presentaron una reducción de la carga viral (ILIB: −1,7 log; CTRL: −2,3 log). En el grupo ILIB, el 61,5% de los participantes alcanzaron una carga viral indetectable (p = 0,449). No hubo aumento en el recuento de LT CD4+ (p = 0,287), pero se observó una reducción no significativa en el recuento de LT CD8+ en el grupo ILIB (p = 0,173). No hubo diferencias estadísticamente significativas entre los grupos para ningún resultado (p > 0,17). El estudio se interrumpió prematuramente durante el análisis provisional.

**Conclusión:**

La ILIB, como terapia adyuvante al TARV, no mostró superioridad clínica ni estadística en los parámetros inmunológicos y virológicos evaluados.

**Registro del ensayo (ReBEC):**

RBR-7j9rv5d (https://ensaiosclinicos.gov.br/trial/10848)

## INTRODUCCIÓN

Aunque existen terapias prometedoras asociadas a la cura del VIH, no hay pruebas suficientes para afirmarlo. Todavía no existe una cura para la infección por el VIH, pero la terapia antirretroviral (TARV) actual es eficaz. El objetivo del tratamiento es inhibir la replicación del virus en el organismo; preservar/restaurar el sistema inmunitario; reducir la probabilidad de aparición de cepas virales más resistentes; disminuir las tasas de morbilidad, mortalidad y hospitalización; disminuir el riesgo de desarrollar enfermedades oportunistas y aumentar la duración y la calidad de vida de las personas que viven con el VIH/síndrome de inmunodeficiencia adquirida (SIDA) (PVV)^(1)^. Este tratamiento tiene un gran impacto en la supervivencia de las PVV, alterando así la caracterización epidemiológica del VIH/SIDA^(1)^.

El Protocolo Clínico y Directrices Terapéuticas para el Manejo de la Infección por VIH en Adultos del Ministerio de Salud afirma que el objetivo del seguimiento y el tratamiento es alcanzar una carga viral (CV) indetectable^(2)^. Sin embargo, incluso con el TARV y el seguimiento, algunas PVV no alcanzan este nivel, lo que puede deberse a una mala adherencia al tratamiento y a la resistencia viral^(3)^.

Una estrategia sugerida para alcanzar una CV indetectable en PVV es el uso de la irradiación intravascular de sangre con láser*,* del inglés *intravenous laser irradiation of blood* (ILIB), como alternativa terapéutica adyuvante.

Desde su creación, los láseres han experimentado una gran evolución tecnológica en los campos de la física y la medicina, y se utilizan en el tratamiento de diversas patologías. Consisten en un medio activo—sólido, líquido o gaseoso—que emite luz al ser excitado por una fuente de energía^(4)^. La introducción de la terapia con láser de baja potencia en 1966 se consolidó como la modalidad terapéutica más investigada en el campo de las aplicaciones clínicas del láser^(4)^.

Originalmente, la ILIB se realizaba mediante una punción venosa en la que se acoplaba el dispositivo. Actualmente, con los avances tecnológicos, se puede realizar de forma transcutánea, cerca de los vasos sanguíneos, es decir, en contacto con la piel, sin necesidad de punción^(5)^. A partir de ello, en 2021 se sugirió que el término utilizado para describir este procedimiento no invasivo fuera «fotobiomodulación vascular»^(6)^. Sin embargo, todavía hay divergencias en la literatura sobre la terminología utilizada, con estudios que se refieren a ella como «ILIB transcutánea»^(7)^, «ILIB modificada»^(8)^, «fotobiomodulación sistémica vascular»^(9)^, entre otros. Por lo tanto, en el presente estudio, considerando que el término ILIB sigue utilizándose con frecuencia, hemos optado por denominar la técnica como ILIB, de manera más generalizada.

La acción de la ILIB actúa modulando la respuesta inmunitaria, estimulando directamente enzimas antioxidantes, como la superóxido dismutasa (SOD), la catalasa y la glutatión peroxidasa^(10)^. Estas enzimas tienen la función de neutralizar los radicales libres presentes en el organismo, reduciendo el estrés oxidativo, que es un factor común en diversas afecciones inflamatorias e infecciosas^(10)^.

En las personas que viven con el VIH, el estrés oxidativo se intensifica por la presencia continua del virus, lo que puede contribuir a la progresión de la enfermedad y a la aparición de comorbilidades^(11)^. Por lo tanto, el ILIB puede actuar como terapia adyuvante al modular el sistema inmunitario y reducir el estrés oxidativo. Este efecto sistémico puede favorecer la preservación de las células T CD4+ y mejorar la respuesta inmunitaria, contribuyendo a la reducción de la progresión viral y de las comorbilidades asociadas.

Los estudios clínicos muestran efectos positivos de la ILIB en la regulación de la presión arterial, la glucosa, los triglicéridos y el colesterol^(12)^, la mejora de la trombocitopenia, la neutropenia y los trastornos de la hemoglobina en pacientes en quimioterapia^(13)^, protección contra el envejecimiento ovárico, mejora de la independencia de pacientes post-ictus^(14)^, mejora de los síntomas en personas con COVID-19 prolongada^(15)^, dolor y trastornos del sueño^(16)^, entre otros.

Sin embargo, solo se ha encontrado un estudio con el uso de ILIB en PVV. En este estudio, la ILIB se utilizó en las regiones de los vasos cubitales, el hígado y el timo, presentando una reducción de los signos y síntomas de PVV y hepatitis, un aumento de los recuentos de linfocitos T-CD4+ (LT-CD4+), linfocitos T-CD8+ (LT-CD8+) y linfocitos totales, y disminución de la CV en participantes con infección por VIH^(17)^. Este estudio se realizó a partir de 1995 en personas que aún no utilizaban TARV. Posteriormente, después de 1998, se introdujo la ILIB como tratamiento para algunas complicaciones de la TARV, como náuseas, intoxicación y deterioro del estado general, con efectos positivos y aumento del recuento de leucocitos^(17)^.

Los autores del estudio creían que la irradiación con *láser* podría interferir en el ciclo celular alterado por la infección, lo que provocaría un aumento de la microcirculación sanguínea y la regeneración de las células dañadas. También se observó que la carga viral plasmática se mantuvo baja durante mucho tiempo después de finalizar el tratamiento con ILIB^(17)^.

Ante la escasez de estudios sobre la ILIB en personas con VIH, el presente estudio tuvo como objetivo evaluar la eficacia de la ILIB en la reducción de la CV y el aumento del recuento de LT-CD4+ y LT-CD8+ en personas viviendo con VIH. La hipótesis del estudio fue que las personas con VIH sometidas a ILIB presentarían una reducción de la CV y un aumento del recuento de LT-CD4+ y LT-CD8+.

## MÉTODO

### Tipode Estudio

Análisis provisional de los datos de un ensayo clínico aleatorizado, paralelo, controlado y simple ciego realizado para verificar la eficacia de la ILIB en la reducción de la CV y el aumento del recuento de LT-CD4+ y LT-CD8+ en PVV. El análisis provisional consiste en comparaciones entre grupos de intervención realizadas antes de la conclusión formal del estudio, generalmente antes de que finalice el reclutamiento. Se utiliza a menudo junto con reglas de interrupción, de modo que un estudio puede interrumpirse si los participantes se exponen a un riesgo innecesario^(18)^. Este estudio se registró en el Registro Brasileño de Ensayos Clínicos (ReBEC: RBR-7j9rv5d – U1111-1262-3868) el 27 de enero de 2021. La recolección de datos se llevó a cabo entre febrero de 2021 y diciembre de 2022.

### Lugar del Estudio

El estudio se realizó en un Servicio de Atención Especializada (SAE) de Infectología en el interior del estado de São Paulo. El SAE de Infectología es un servicio público que forma parte del Sistema Único de Salud (SUS), que ofrece asistencia multidisciplinar a personas que viven con el VIH, hepatitis crónicas por virus B y C, personas con infección por HTLV-I/II y víctimas de accidentes (laborales o sexuales) con riesgo biológico de contraer infecciones por los agentes etiológicos mencionados. Durante el período del estudio, el servicio atendía a cerca de 1000 PVV.

### Población

Se invitó a participar en la investigación a todas las PVV que recibían atención ambulatoria, tenían CV detectable y seguían el mismo esquema terapéutico de TARV.

### Criterios de Selección

Se incluyeron PVV de ambos sexos, con edad ≥ 18 años, en tratamiento ambulatorio, con CV detectable y tratados con tenofovir + lamivudina + dolutegravir (independientemente del tiempo de tratamiento). Se excluyeron las mujeres embarazadas, las personas con glaucoma, las personas institucionalizadas y aquellas que no pudieron completar el formulario debido a un deterioro cognitivo.

### Definición de la Muestra y Análisis Provisional

Teniendo en cuenta la hipótesis clínica de que se produce una mejora superior a 0,5 log de la carga viral en el grupo tratado, con un 5% de *abandono*, un alfa del 5% (unicaudal) y una potencia del 80%, se estimó una muestra de 86 participantes (43 participantes en cada grupo de tratamiento).

Debido a la ausencia de otros estudios sobre ILIB que presentaran referencias sobre la reducción de la carga viral y el aumento de LT-CD4+ y LT-CD8+ en PVV, se optó por realizar un análisis provisional que se llevó a cabo tras 28 tratamientos. En ese momento, el estudio se interrumpió (por futilidad), ya que no se identificó una media de 0,5 log de carga viral superior en el grupo de intervención.

### Recolección de Datos

El presente estudio incluyó a 28 participantes, de los cuales 24 acudieron al ambulatorio y cuatro recibieron visitas domiciliarias de acuerdo con los criterios de inclusión y exclusión.

Los investigadores involucrados cuentan con cursos de formación en laserterapia y recibieron capacitación para la estandarización de la aplicación del láser en el estudio. La investigadora principal (enfermera) realizó una consulta de enfermería a ambos grupos que participaron en la investigación. El primer día se aplicó un cuestionario sociodemográfico y se les preguntó sobre el tiempo de diagnóstico, enfermedades oportunistas presentes o pasadas, otros medicamentos utilizados y el número de veces que se habían olvidado de tomar el TARV en un mes. También se aplicó un cuestionario el primer y el último día de las sesiones para evaluar la incidencia de náuseas, vómitos, debilidad, sudoración, insomnio, falta de apetito, diarrea u otros síntomas.

Todos los participantes recogieron las pruebas (carga viral, LT-CD4+ y LT-CD8+) 10 días antes del inicio del tratamiento, ya que es el tiempo que tarda en estar disponible el resultado en el Sistema de Control de Exámenes Laboratorios (Siscel) de la Red Nacional.

Todas las muestras se recogieron mediante punción venosa en los miembros superiores de los participantes y se almacenaron en tubos con ácido etilendiaminotetraacético (EDTA). Las muestras se procesaron en el equipo Abbott M2000SP/M2000RT con extracción, amplificación y detección del material en el laboratorio del hospital de referencia de la institución educativa.

Los participantes seleccionados fueron aleatorizados en dos grupos: grupo ILIB y grupo control (CTRL). La aleatorización de los bloques fue realizada por una persona externa y la secuencia de aleatorización se llevó a cabo mediante un *software* informático.

Al tratarse de un estudio simple ciego, los participantes no fueron informados sobre el tratamiento que recibieron. El análisis de los datos fue realizado por una tercera persona que desconocía la asignación de los grupos.

En un entorno cómodo, se invitó a los participantes a sentarse en una silla o tumbarse en una camilla, según sus preferencias. Se midieron la presión arterial, la frecuencia cardíaca y la oximetría antes y al final de todas las sesiones, como parámetro de seguridad para los efectos sistémicos de la luz. No se observaron cambios significativos.

Tanto los participantes como los investigadores utilizaron gafas protectoras. Los investigadores tomaron el pulso radial y se colocó una pulsera con un dispositivo acoplado en la muñeca.

El dispositivo fue calibrado y configurado por la empresa colaboradora para dos funciones únicamente: control e intervención. Para la función de intervención se activó la luz roja, con emisión de sonido y un cronómetro de 30 minutos que se mostraba en la pantalla del dispositivo. Para la función de control, la luz no se activó, y en la pantalla del dispositivo solo se mostraban el sonido y el cronómetro.

El grupo ILIB recibió tratamiento con láser durante 30 minutos diarios durante 10 días, excepto sábados, domingos y festivos. Tras el primer ciclo, los participantes descansaron durante 20 días; a continuación, el tratamiento se continuó durante 10 días más. El grupo CTRL se sometió al mismo régimen, pero utilizando la técnica de radiación sin láser. El protocolo utilizado se adaptó del protocolo ILIB tradicional para tratamientos sistémicos, disponible en la aplicación de la empresa proveedora del dispositivo (DMC Protocolos). En el protocolo original, se recomienda una nueva pausa de 20 días, seguida de 10 días más de sesiones. Sin embargo, optamos por seguir un estudio anterior que aplicó un protocolo adaptado y obtuvo resultados positivos^(13)^. Todos los participantes utilizaron el mismo protocolo para el estudio. Las sesiones se estandarizaron para el período de la mañana, con cita previa, y se realizaron entre las 8:00 y las 10:00 en todos los participantes.

Al final de los últimos 10 días de la ILIB, se recogieron muestras de ambos grupos para pruebas de CV, LT-CD4+ y LT-CD8+; se volvió a aplicar el cuestionario utilizado para evaluar las quejas y las diferencias de perspectiva de los participantes desde el inicio hasta el final del periodo de tratamiento. Este cuestionario fue autoadministrado o administrado por una tercera persona que no formaba parte del estudio para evitar sesgos en las respuestas.

Todos los participantes completaron 20 sesiones de ILIB. Los parámetros de irradiación utilizados en este estudio fueron: Equipo: Therapy Ilib, DMC Equipamentos®; Fuente de luz: láser (diodo); Longitud de onda: 660 nm; Potencia: 100 mW; Modo de funcionamiento: continuo; Área del *«punto»*: 0,0984 cm^2^; Tiempo de irradiación: 30 minutos; Energía total: 180 J; Densidad energética: 1,830 J/cm^2^ o 1,83 KJcm^2^; Lugar de irradiación: pulso; Vaso sanguíneo: arteria radial; Acoplamiento: contacto con la piel; Frecuencia de la sesión: 10 días continuos, intervalo de 20 días y luego 10 días más.

Todos los materiales utilizados (equipo, pulsera, gafas protectoras y almohadilla) se desinfectaron al final de cada sesión y entre cada participante, utilizando algodón y alcohol al 70%, según las recomendaciones del fabricante del equipo.

### Análisis de Datos

Las variables se almacenaron en una hoja de cálculo *Excel* y se analizaron descriptivamente mediante el *oftware* de I*nternational Business Machines Corporation* (IBM) – *Statistical Package for the Social Sciences* (SPSS) 25.0.

Se utilizaron pruebas de tendencia chi-cuadrado para categorizar las variables, cuyas ocurrencias se evaluaron en cada grupo y en cada momento.

Se utilizó la prueba *U* de *Mann-Whitney* para comparar los datos iniciales entre los dos grupos, y se utilizó un modelo lineal generalizado de efectos mixtos con regresión gamma para la comparación longitudinal entre los dos grupos y para estimar los tamaños de los efectos.

### Aspectos Éticos

El presente estudio fue aprobado (Dictamen: 4.419.222) por el Comité de Ética en Investigación (CEP) de la Facultad de Medicina de Botucatu y los procedimientos se llevaron a cabo siguiendo todas las normas éticas de conformidad con la Resolución n.º 466, de 12 de diciembre de 2012^(19)^. Todos los participantes firmaron el Término de Consentimiento Libre y Esclarecido antes de comenzar a participar en la investigación.

El dispositivo utilizado durante el estudio fue donado y la empresa patrocinadora no interfirió en ningún momento en los resultados de esta investigación.

## RESULTADOS

Se reclutaron un total de 28 participantes (grupo ILIB: 13 y grupo CTRL: 15). El diagrama de flujo según las *Normas Consolidadas para la Presentación de Ensayos Clínicos* (CONSORT)^(20)^ se muestra en la Figura 1.

**Figura 1:** Flujograma CONSORT de los procedimientos de inclusión, asignación, seguimiento y análisis de la muestra de la investigación.

* Mujeres embarazadas, personas con glaucoma, personas institucionalizadas o con déficit cognitivo; ^†^ = Debido al trabajo o a la falta de transporte.

Las consultas se realizaron en un SAE de Infectología vinculado al SUS. La totalidad de los participantes eran usuarios exclusivos del sistema público de salud, lo que puede atribuirse al hecho de que las PVV con acceso a planes de salud tienden a buscar atención en servicios privados. Este aspecto ayuda a explicar el perfil sociodemográfico observado en la muestra, caracterizado por un bajo nivel educativo y bajos ingresos. Los datos demográficos se presentan en la Tabla 1.

**Tabla 1:** Distribución de los pacientes según características sociodemográficas. Botucatu – SP, Brasil, 2021–2022.

Variables	Grupo ILIB n (%)	Grupo CTRL n (%)	Total – n (%)
Sexo			
Masculino	9 (32,1)	11 (39,3)	20 (71,4)
Femenino	4 (14,3)	4 (14,3)	8 (28,6)
Edad	46 (24–64)*	38,2 (18–64)*	42 (18–64)*
Color			
Blanca	11 (39,3)	10 (35,7)	24 (75)
Parda	2 (7,1)	3 (10,7)	5 (17,9)
Negra	0 (0)	2 (7,1)	2 (7,1)
Estado Civil			
Soltero	8 (28,6)	11 (39,3)	19 (67,9)
Casado	2 (7,1)	12 (7,1)	4 (14,3)
Divorciado	2 (7,1)	1 (3,6)	3 (10,7)
Unión libre	1 (3,6)	1 (3,6)	2 (7,1)
Escolaridad			
Primario Incompleto	4 (14,3)	7 (25)	11 (39,3)
Secundario Incompleto	5 (17,9)	5 (17,9)	10 (35,7)
Secundario Completo	1 (3,6)	3 (10,7)	4 (14,3)
Superior	3 (10,7)	0 (0)	3 (10,7)
Ingreso Familiar (Per cápita)	R$ 1.491,67 (275,00–5.000)*	R$803,21 (166,66–2.500,00)*	R$1.122,85 (166,66–5.000)*
Procedencia			
DRS** – Bauru			28 (100)

*Media (Mínimo – Máximo)

**DRS – Departamento Regional de Salud

La Tabla 2 muestra los motivos de consulta que los participantes presentaron el primer y el último día del tratamiento. Se observó una mejora en todos los motivos de consulta relatados, pero no fue estadísticamente significativa.

**Tabla 2:** Cuestionario inicial y final referente a los motivos de consulta (n = 28) – Botucatu, SP, Brasil, 2021–2022.

Motivos de consulta	Antes n (%)	Después n (%)
Náusea		
Si	5 (17,8)	1 (3,6)
Vómito		
Si	1 (3,6)	0 (0)
Debilidad		
Si	15 (53,6)	5 (17,9)
Transpiración		
Si	5 (17,9)	1 (3,6)
Insomnio		
Si	13 (46,4)	2 (7,1)
Inapetencia		
Si	6 (21,4)	2 (7,1)
Diarrea		
Si	3 (10,4)	1 (3,6)

No se observaron diferencias en las características basales de los participantes. La Tabla 3 presenta las 20 sesiones previas y posteriores a las intervenciones. No se observaron diferencias entre los grupos en relación con LT-CD4+, LT-CD8+ y CV tras las intervenciones (p > 0,17). Se observó una reducción no significativa en los valores de LT-CD8+ en el grupo ILIB al final del tratamiento. Ambos grupos redujeron la CV, y el 40% de los participantes del grupo CTRL y el 61,5% del grupo ILIB alcanzaron una CV indetectable al final del tratamiento (p = 0,449).

**Tabla 3:** Valores de LT-CD4+, LT-CD8+ y CV antes y después del tratamiento (n = 28) – Botucatu, SP, Brasil, 2021–2022.

	Tiempo	Grupo		Dif (IC 95%)^‡^	p-valor
		ILIB* $\bar{x}$ (*s*)	CTRL $\bar{x}$ (*s*)		
LT-CD4+	D0	346 (204)	452 (353)	−7 (−199–186)	0.944
	D20	403 (239)	520 (338)	−117 (−336–102)	0.287
	Dif (CI 95%)	57 (−129–242)	181 (−44–406)		
LT-CD8+	D0	981 (667)	838 (374)	55 (−330–440)	0.775
	D20	918 (361)	1,119 (337)	−197 (−483–89)	0.173
	Dif (CI 95%)	74 (−278–424)	215 (−111–542)		
CV^§^ (Log)	D0	2.8 (1.2)	3.7 (1.3)	−0.8 (−1.8–0.1)	0.086
	D20	1.1 (1.4)	1.4 (1.5)	−0.3 (−1.4–0.8)	0.597
	Dif (CI 95%)	−1.7 (−2.8– −0.7)	−2.3 (−3.3– −1.3)		

*ILIB = *intravenous laser irradiation of blood*

^†^CTRL = control

^‡^Dif (IC 95%) = Diferencia e intervalo de confianza entre D0 para D20, o ILIB para CTRL

^§^CV = carga viral

La Tabla 4 muestra la evaluación individual relacionada con el aumento o la disminución de los valores de LT-CD8+ y con carga viral indetectable al final del protocolo. Se observó que de los siete pacientes que presentaron una disminución de LT-CD8+ en el grupo ILIB, cinco tenían una carga viral indetectable y, de estos cinco, tres presentaban enfermedades oportunistas durante el tratamiento.

**Tabla 4:** Distribución de los participantes del estudio en relación con LT+CD8+, carga viral indetectable y enfermedad oportunista (n = 28) – Botucatu, SP, Brasil, 2021–2022.

		LT-CD8+	
	n	CV* indetectable	Enfermedad oportunista
**CTRL^†^**	**15**	**6 (40%)**	–
Aumento LT-CD8+	11	4	
Reducción LT-CD8+	4	2	
**ILIB^‡^**	**13**	**8 (61,5%)**	
Aumento LT-CD8+	6	3	
Reducción LT-CD8+	7	5	3
**Total general**	**28**		

*CV = carga viral

^†^CTRL = control

^‡^ILIB = *intravenous laser irradiation of blood*

Ante los resultados obtenidos y la inviabilidad de continuar con el protocolo de tratamiento, el estudio se interrumpió prematuramente durante el análisis provisional.

## DISCUSIÓN

Hasta el momento, este ha sido el primer ensayo clínico aleatorizado, paralelo, controlado y simple ciego que ha verificado la eficacia de la ILIB, utilizada junto con la TARV, en la reducción de la CV y el aumento del recuento de LT-CD4+ y LT-CD8+ en PVV. Después de 20 sesiones, no hubo diferencias significativas en los cambios de LT-CD4+, LT-CD8+ y CV entre los grupos ILIB y CTRL.

En nuestro estudio, se observó que la mayoría de los participantes del grupo ILIB que presentaron una reducción en el recuento de LT-CD8+ tenían una carga viral (CV) indetectable. Sin embargo, también desarrollaron enfermedades oportunistas durante el tratamiento, lo que puede justificar la disminución de los LT-CD8+. Estudios anteriores sugieren que factores como la edad avanzada, los recuentos bajos de LT-CD4+ al inicio de la terapia antirretroviral o el uso de drogas inyectables pueden estar asociados con una respuesta inmunológica inadecuada^(21–23)^. Además, las vacunas pueden interferir temporalmente en los niveles de LT-CD4+, que tienden a normalizarse entre dos y seis semanas después de la administración^(24)^.

Se espera que la CV sea indetectable en los seis meses siguientes al inicio del TARV^(25)^. En el presente estudio, la mayoría de los participantes de ambos grupos presentaron una CV indetectable al final del tratamiento.

Algunas personas no alcanzan una CV indetectable, lo que puede deberse a la falta de adherencia al tratamiento o al fracaso virológico^(3)^. Una de las razones de la falta de adherencia al tratamiento es la aparición de reacciones adversas relacionadas con la TARV, como náuseas, vómitos, cefalea, insomnio, entre otras^(26)^. En el presente estudio, las PVV informaron una mejora de las quejas generales tras la terapia con ILIB.

También se evidenció en el grupo CTRL una mejora de la debilidad y del sueño. Estos resultados observados en el grupo no tratado pueden atribuirse a las expectativas de los participantes, a la experiencia del tratamiento y al hecho de haber recibido atención^(27)^. Muchos factores pueden afectar los resultados del tratamiento, la calidad de la atención, la memoria, el lugar donde se realizó el tratamiento y la interacción entre el paciente y el profesional^(28–30)^.

La Sociedad para los Estudios Interdisciplinarios del Placebo *(Society for Interdisciplinary Placebo Studies)* definió los «efectos» del placebo como cambios específicos derivados del régimen placebo, tales como mecanismos de expectativa neurobiológicos y psicológicos; sin embargo, la «respuesta» al placebo se definió como cambios en la salud del paciente como resultado del uso del placebo, con regresión del curso natural de la enfermedad^(31)^.

Este estudio presenta algunas limitaciones. En primer lugar, se enfrentaron dificultades en el reclutamiento de participantes elegibles debido al protocolo adoptado, que consistía en 10 sesiones realizadas en días consecutivos, seguidas de una pausa de 20 días y, posteriormente, otras 10 sesiones consecutivas, lo que imposibilitó la participación de muchas PVV. Cabe recordar que la renta per cápita de los participantes era de 1122,85 reales, lo que pone de manifiesto la vulnerabilidad social del grupo.

Aunque este protocolo es recomendado por la empresa proveedora del dispositivo para actuar sobre el sistema inmunológico, resultó inviable para la realidad del presente estudio. Por otro lado, investigaciones que emplearon la ILIB con una frecuencia de dos sesiones semanales reportaron efectos positivos^(12–32)^, lo que puede representar una alternativa viable para mejorar la adherencia y aumentar el número de participantes en un estudio futuro. Cabe destacar que no se adoptó un protocolo estandarizado basado en el biotipo corporal de los participantes. Además, la literatura aún carece de recomendaciones específicas que relacionen el biotipo corporal con la definición del tiempo de aplicación y la dosimetría, siendo los estudios existentes inconclusos^(33,34,35)^.

En segundo lugar, el número de individuos con CV detectable en la muestra fue relativamente pequeño, ya que la mayoría de las personas inician la TARV poco después del diagnóstico y presentan CV indetectable. Además, los participantes con CV detectable a menudo ya presentaban una baja adherencia al TARV, lo que también repercutió negativamente en la adherencia al protocolo del estudio.

Por último, otra limitación fue que no se preguntó a los participantes, en todas las sesiones, sobre la presencia de síntomas indicativos de infección o antecedentes recientes de vacunación. Estos factores pueden haber contribuido a la reducción de los recuentos de LT-CD4+ y LT-CD8+ observada en algunos casos, lo que constituye una posible variable de confusión en los resultados. Sin embargo, tampoco se evaluó si las participantes de sexo biológico femenino se encontraban en diferentes fases del ciclo menstrual durante el tratamiento. Los estudios indican que las fluctuaciones hormonales a lo largo del ciclo pueden modular la inmunidad, con evidencia de una reducción transitoria en el recuento de linfocitos T durante la menstruación, especialmente en mujeres VIH positivas, lo que puede influir en la respuesta inmunológica y en la interpretación de los parámetros de laboratorio^(36)^.

Por otro lado, como contribuciones del presente estudio, se sugieren protocolos aplicables en contextos clínicos, con la adopción de enfoques con un menor número de sesiones y evaluaciones clínicas más detalladas de los participantes en cuanto a cualquier síntoma de infección, estado vacunal o falta de respuesta inmunológica para justificar un mejor control de los recuentos de LT-CD4+ y LT-CD8+.

## CONCLUSIÓN

No hubo diferencias en los valores de LT-CD4+, LT-CD8+ y carga viral en PVV que recibieron ILIB como tratamiento adyuvante al TARV.

Sin embargo, los participantes tratados que presentaron una reducción en el recuento de CD8+ presentaron CV indetectable.

Teniendo en cuenta la escasez de estudios clínicos que han evaluado a PVV sometidas a ILIB, se entiende que la realización de otros estudios con protocolos aplicables en contextos clínicos con un menor número de sesiones, evaluaciones clínicas más detalladas de los participantes y una muestra más amplia podría aportar resultados significativos.

## Data Availability

El conjunto completo de datos que respalda los resultados de este estudio está disponible previa solicitud a la autora de correspondencia (Fabiana Tomé Ramos). El conjunto de datos no está disponible públicamente, ya que contiene información que podría comprometer la privacidad de los participantes del estudio.
